# Association Between Trust in Health Care Professionals and Health Care Access: Insights From an Online Survey Across 21 Countries

**DOI:** 10.3389/ijph.2025.1607884

**Published:** 2025-04-10

**Authors:** Louisa Ewald, John Bellettiere, Tamer H. Farag, Kristina M. Lee, Sidhartha Palani, Emma Castro, Amanda Deen, Catherine W. Gillespie, Bethany M. Huntley, Alison Tracy, Anna-Carolina Haensch, Frauke Kreuter, Wiebke Weber, Stefan Zins, Wichada La Motte-Kerr, Yao Li, Kathleen Stewart, Emmanuela Gakidou, Ali H. Mokdad

**Affiliations:** ^1^ Institute for Health Metrics and Evaluation, University of Washington, Seattle, WA, United States; ^2^ Meta, Menlo Park, CA, United States; ^3^ Institute for Statistics, Ludwig-Maximilian Universitat, Munich, Germany; ^4^ Department of Geographical Sciences, University of Maryland, College Park, MD, United States; ^5^ Institute for Employment Research of the German Federal Employment Agency, Nuremberg, Germany; ^6^ Department of Health Metrics Sciences, University of Washington, Seattle, WA, United States

**Keywords:** trust in health care providers, health care access, health care delays, COVID-19 pandemic, health systems, health care utilization, trust in institutions

## Abstract

**Objectives:**

This study evaluates the association between trust in health care professionals and health care delays across 21 countries.

**Methods:**

We apply logistic regression models to survey data of over 621,000 individuals collected in Spring 2023.

**Results:**

Results show 44.5% of respondents with medical conditions experienced delays in accessing health care and 44.1% reported lack of trust in health care professionals. Those who trusted health care professionals had significantly lower odds of delaying medical care. Trust was most strongly associated with delays in the United Kingdom (OR = 0.373, 95% CI = 0.273–0.510), while South Africa had the smallest association (OR = 0.762, 95% CI = 0.582–0.997).

**Conclusion:**

Trust is important in influencing health care-seeking behaviors, though the causal direction warrants further research. There is a need for targeted strategies to build and sustain trust in health care relationships as well as enhancing health care access.

## Introduction

Health outcomes are determined by a complex web of factors, with two important elements being timely access to care and the trust between patients and health care providers. Timeliness of care plays a large role in preventing and reducing the risk of complications, and delays in care are associated with an increased risk of mortality for conditions such as cancer, tuberculosis, and maternal health [1–3]. Additionally, COVID-19 negatively impacted the delivery of care in many health systems across the globe [4–10].

Trust is a nuanced construct, generally defined where the truster believes and optimistically accepts that the trustee will care for the truster’s interests in a vulnerable situation [11]. Previous studies have found the trust between patients and their health care providers can significantly influence health outcomes and patient satisfaction [12–14]. In particular, trust in health care providers influences a wide range of outcomes from treatment adherence to vaccine uptake, and plays a pivotal role in seeking care and subsequent health outcomes [11, 15]. Higher levels of trust in health care professionals are associated with fewer reported symptoms and greater satisfaction with care [15]. Furthermore, trust extends beyond individual providers to encompass health institutions and has been shown to correlate with compliance with medical advice and vaccine acceptance [16].

The theoretical underpinnings of trust’s influence on health care-seeking behaviors draw from various health behavior theories. According to the Health Belief Model, individuals are more likely to engage in health-seeking behaviors if they believe that a particular health action would reduce their susceptibility to, or severity of, a disease [17]. Trust in health care professionals can enhance this belief by reinforcing the perceived benefits of seeking care and mitigating perceived barriers.

While previous research has highlighted the potential impact of trust on health care utilization, the specific mechanisms through which trust may influence access to necessary health care services are not fully developed. The relationship between trust and health care access is potentially bidirectional, with limited access or perceived low quality of care diminishing trust, which in turn, may discourage individuals from seeking care.

The COVID-19 pandemic has brought the issue of trust in health care systems to the forefront with increasing calls to understand this relationship [18]. Between 2015 and 2019, trust in the safety and effectiveness of vaccines fell in many parts of the world [19]. However, during the pandemic, physicians and nurses were reported as the most trusted sources of health information, a testament to the critical role of health professionals in times of uncertainty [20]. It is important, now more than ever, for health officials and governments to better understand how trust influences health care access and outcomes within their own health care systems.

Timely and relevant data on an individual’s level of trust in health care professionals and their subsequent health care access is severely limited. Online surveys, including those leveraging social media platforms, provide valuable insights into topics such as trust, from an individual’s perspective [21].

Given the scarcity of timely and relevant data on an individual’s level of trust in health care professionals and their subsequent health care access, our study aims to bridge this gap. Using data from the Pandemic Recovery Survey (PRS), conducted across 21 countries with over 621,000 respondents, we examine the relationship between trust in health care professionals and health care access delays. By understanding how trust influences health care access, our study seeks to inform policies that improve health outcomes and address the complex interplay between trust, access, and quality in health care systems.

## Methods

### Study Design and Participants

This cross-sectional, internet-based survey was conducted as part of the Pandemic Recovery Survey (PRS) across 21 countries from March to May 2023 [22]. The PRS sought to capture the societal and population-level consequences of the COVID-19 pandemic, focusing on economic, educational, and health outcomes. The target population included active Facebook users aged 18 and over in the 21 selected countries. Countries were selected based on region, population, existing health care systems, and Facebook availability and usage.

The questionnaire was translated and checked by native speakers in 15 languages and pilot tested in all countries before launch. All materials and procedures for this study were reviewed and approved by the University of Washington Institutional Review Board (STUDY00016693).

### Sampling Methodology

We randomly sampled the Facebook Active User Base stratified by gender for each country. Respondents were invited via Facebook and redirected to the Qualtrics platform for survey completion. Participants were not able to take the survey twice or send the link to others. All participants provided informed consent prior to taking the survey. Meta did not have access to the data [9].

The weighting methodology encompassed two steps: inverse propensity score weighting to address non-response bias and raking weights to align with known population totals. The weights were scaled to population estimates from IHME, used in the Global Burden of Disease study, and calibrated across demographic cross-classifications within each country’s observations [23]. The study design and questionnaire were submitted and approved by the Institutional Review Board of the University of Washington.

### Measures

Questions regarding health care delays were asked to respondents who reported an existing medical condition (heart attack, stroke, high blood pressure, cancer, diabetes, lung disease, dementia or Alzheimer’s disease, mental health condition, addiction or substance use disorder, liver disease, and kidney disease) and currently needed care. Respondents were then asked if they had received care from a health care provider for their condition(s) in the previous 6 months. Respondents were able to answer that they had received care when needed, only received care some of the times needed, did not receive care when needed, or did not require care or treatment. In our analysis, among respondents who currently need care, delayed care was coded as 1 for respondents not receiving care every time they needed it in the last 6 months and coded as 0 for respondents who received the care they needed.

Respondents were asked whether they felt health care professionals (such as physicians and nurses) were “very trustworthy, somewhat trustworthy, neither trustworthy nor untrustworthy, not very trustworthy, or not trustworthy at all.” Respondents were also asked about their trust in other institutions such as the national government, international health organizations, the local police, and community leaders. These questions was derived from indices used in similar contexts to assess the level of trust in various institutions [9, 24, 25]. Those reporting that they considered health care professionals very or somewhat trustworthy were categorized into the trusts health care professionals category while the other three were categorized as not trusting health care professionals. We examined interactions between each institution and delayed care, and for the purposes of this study we chose to only include the most relevant variable, trust in health care professionals.

Covariates included age (18–24 years, 30–49 years, and 50 years and older), gender (male, female), educational attainment (primary or less, secondary, college or more), and financial stability (easy to afford household expenses, somewhat difficult, or very difficult).

### Statistical Analysis

We analyzed the data using R software and the survey package [26, 27]. We performed weighted descriptive analyses and cross-tabulations to assess the association between trust in health care professionals and health care access delays. We also employed multilevel logistic regression using the glmer function from the lme4 package, with individuals nested within countries [28]. The model included country, age, gender, education level, trust in health care professionals, and financial stability. Random effects were summarized with variance components, and country-specific random effects were reported relative to the global intercept.

We estimated fixed effects to understand the association of each variable with the likelihood of delayed care, while random effects captured the variation across countries. The model’s fixed effects estimates were exponentiated to obtain odds ratios (ORs), with corresponding 95% confidence intervals (CIs) and p-values, to increase interpretability.

This analysis closely examined the relationship between trust in health care professionals and delays in accessing health care, while controlling for socio demographic covariates. The intent was not to draw independent associations between these covariates and delayed health care access, but instead focus on understanding how trust– or lack thereof– in health care professionals is associated with the likelihood of experiencing delays in care, with other variables adjusting for potential confounders.

## Results

### Sample Characteristics

**FIGURE 1:**
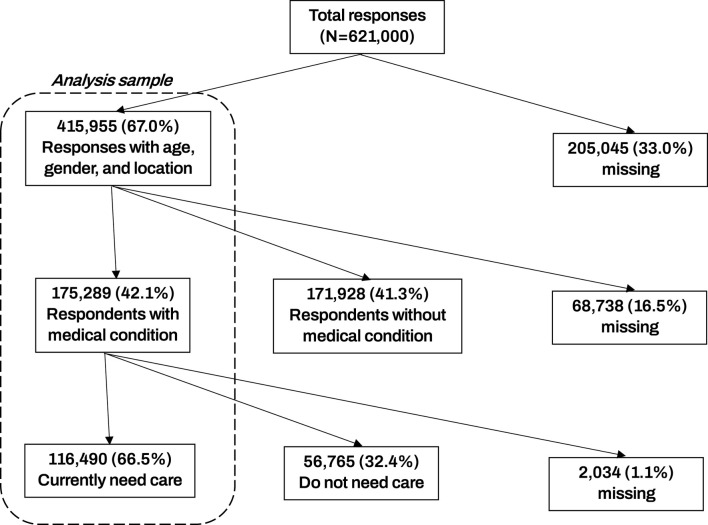
Shows a Strobe diagram of the analysis sample. Among 621,000 total responses, 116,490 contained relevant data and currently needed health care for an existing medical condition. Data are from the Pandemic Recovery Survey 2023, 21 countries.

The Pandemic Recovery Survey (PRS) received over 621,000 individual responses from 21 countries, with 51.8% total male respondents. Additional sample details can be found in Figure 1. The age distribution was as follows: 34.1% aged 18–29 years, 45.3% aged 30–49 years, and 20.6% aged 50 years and older. Educational attainment varied, with 10.0% having primary education or less, 44.0% with secondary or trade school diplomas, and 46.0% with college or higher education (Table 1).

**TABLE 1:** Overall survey characteristics stratified by whether respondent trusts health care professionals or not. Data are from the Pandemic Recovery Survey, 2023 across 21 countries.

Variable	Lacks trust in health care professionals	Trusts health care professionals
Total n	134,266	169,659
Age (%)		
18–29 years	40,005 (29.8)	55,827 (32.9)
30–49 years	63,846 (47.6)	77,089 (45.4)
50+ years	30,415 (22.7)	36,743 (21.7)
Gender (%)		
Female	65,593 (48.9)	78,105 (46.0)
Male	66,613 (49.6)	89,762 (52.9)
Prefer not to answer or non-binary	2,060 (1.5)	1,792 (1.1)
Education (%)		
College or more	56,737 (42.3)	86,125 (50.8)
Primary school or less	13,276 (9.9)	13,498 (8.0)
Secondary school	64,253 (47.9)	70,036 (41.3)
Finances are not easy (%)	106,747 (89.7)	121,751 (80.4)

### Health Care Delays and Trust in Health Care Professionals

Our study found that 44.5% of respondents with medical conditions experienced obstacles in accessing needed health care. Delays were most pronounced in Viet Nam (73.3%), followed by the Philippines, Peru, and Indonesia. Conversely, Japan reported the lowest rate of delayed care, with only 12.8% of respondents indicating such delays.

**FIGURE 2:**
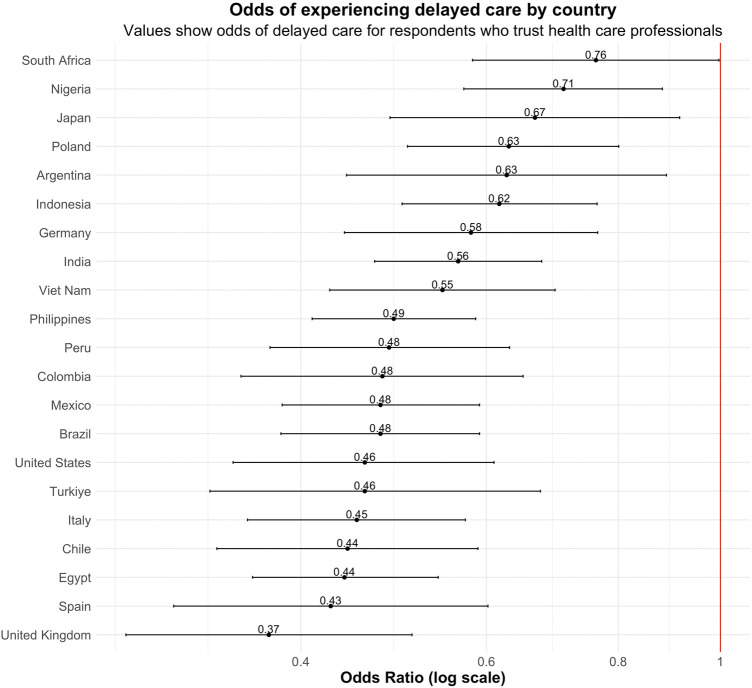
Displays the proportion of respondents in each country who felt health professionals were very trustworthy, not trustworthy, or neither. Data are from the Pandemic Recovery Survey 2023, 21 countries.

Trust in health care professionals varied substantially across the study population, with 44.1% of all respondents expressing lack of trust, as displayed in Figure 2 by country. India reported the highest trust levels, with 67.8% of respondents viewing health care professionals as somewhat or very trustworthy, 26.4% saying health care professionals were neither trustworthy nor untrustworthy, and 5.7% saying they were not trustworthy. In contrast, Peru had the highest proportion of respondents (18.6%) perceiving health care professionals as not trustworthy. Respondents in Indonesia reported the smallest proportion who felt health care professionals were not trustworthy (2.1%), and the largest proportion who felt they were neither trustworthy nor untrustworthy (60.1%). More detailed findings are reported in Supplementary Table S1.

### Odds of Delayed Care

Survey respondents who reported trusting health care professionals were associated with significantly lower odds of experiencing delayed care, after adjusting for age, gender, educational attainment, and financial stability, compared to respondents who lacked trust in health care professionals. When testing for between-country variability, we found that the relationship between trust and delayed care significantly varied by country (p < 0.015).

**FIGURE 3:**
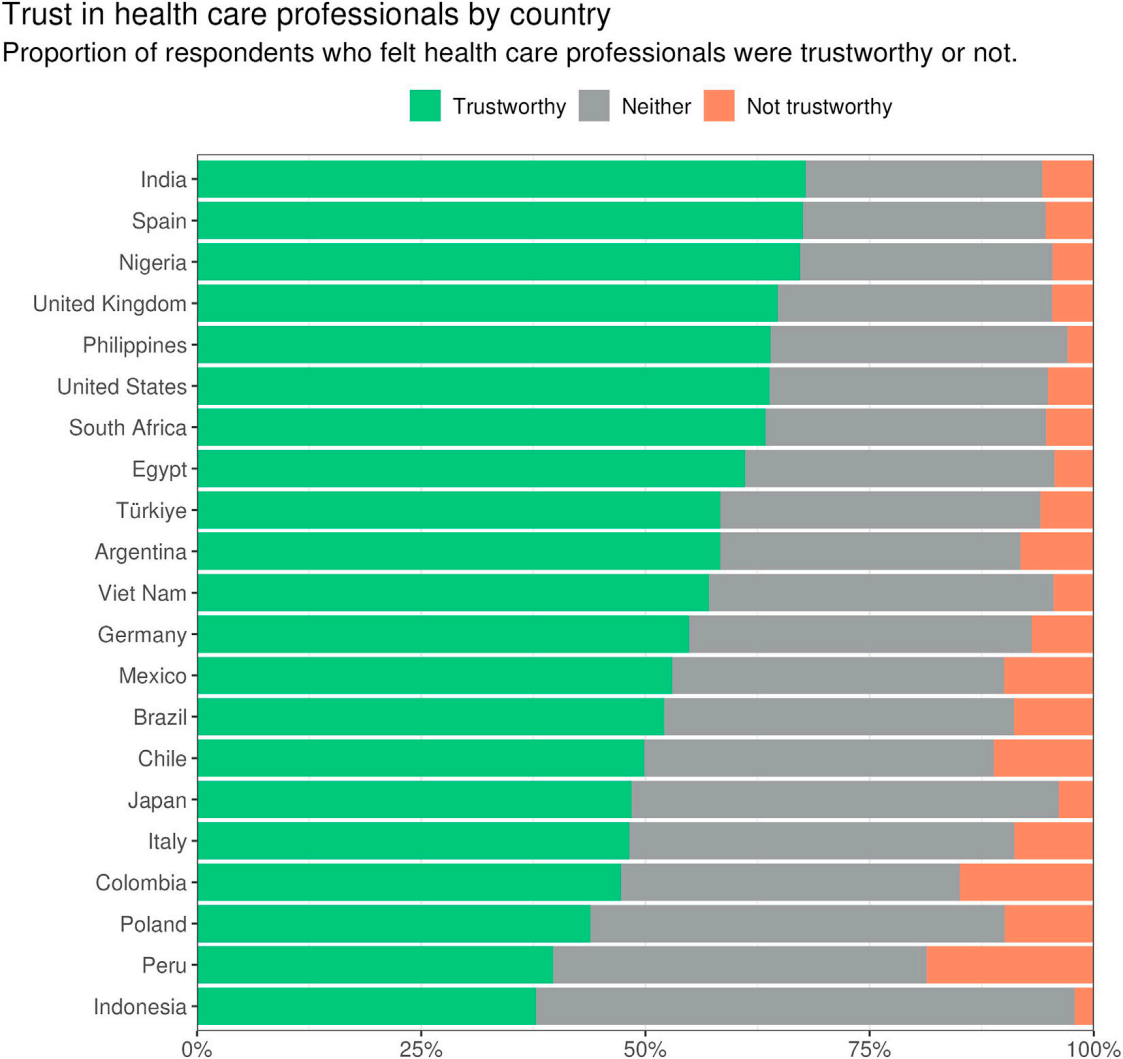
This forest plot shows the results from a multivariable analysis of trust as a predictor of health care delays. Values represent the odds ratios for experiencing delayed health care based on their trust in health care professionals, adjusted for demographic variables including gender, age, education, and financial status. Data are from the Pandemic Recovery Survey 2023, 21 countries.

Figure 3 visually represents the results of the multivariable analysis examining the relationship between trust in health care professionals and the likelihood of experiencing delayed health care across 21 countries. Each line represents a country included in the analysis, with the odds ratio (OR) depicted on the x-axis. An OR less than 1 suggests that trust in health care professionals is associated with reduced delays in accessing health care services.

Results show a wide variation between countries, though all fall under an OR of 1, indicating trust having a protective association with these associations across countries. The United Kingdom showed the largest association (OR = 0.373, 95% CI = 0.273–0.510), while South Africa had the lowest (OR = 0.762, 95% CI = 0.582–0.997).

In the Americas, results were very similar for Brazil (OR = 0.476, 95% CI = 0.383–0.591), Mexico (OR = 0.476, 95% CI = 0.384–0.591), Colombia (OR = 0.478, 95% CI = 0.351–0.650), and Peru (OR = 0.485, 95% CI = 0.374–0.631). Chile displayed the strongest association in the Americas (OR = 0.443, 95% CI = 0.333–0.589) while Argentina had a weaker association (OR = 0.627, 95% CI = 0.442–0.889).

In Europe, the UK, Spain (OR = 0.427, 95% CI = 0.303–0.602), and Italy (OR = 0.452, 95% CI = 0.356–0.573) displayed strong associations between trust and reduced health care delays, while sub-Saharan African countries, represented by Nigeria (OR = 0.710, 95% CI = 0.571–0.881) and South Africa, showed more modest effects of trust on health care access.

Countries in Asia showed similar associations between trust and health care access. The Philippines (OR = 0.490, 95% CI = 0.410–0.586), Viet Nam (OR = 0.545, 95% CI = 0.426–0.697), India (OR = 0.564, 95% CI = 0.470–0.677), Indonesia (OR = 0.617, 95% CI = 0.499–0.764), and Japan (OR = 0.667, 95% CI = 0.486–0.915) all show moderate associations between trust and health care access.

## Discussion

The comprehensive data from the Pandemic Recovery Survey (PRS) across 21 countries provides an opportunity to understand the nuanced relationship between trust in health care professionals and health care access during the later stage of the COVID-19 pandemic. The findings underscore the variance in trust levels across different regions and the corresponding impact on health care delays, with substantial implications for public health systems seeking to improve patient outcomes and satisfaction in the post-pandemic era.

Overall, respondents who expressed trust in health care professionals were associated with significantly lower odds of experiencing delays in health care. Notably, the United Kingdom exhibited the strongest association between high trust levels and reduced delays in accessing health care. This suggests that in contexts where health care systems are perceived as more reliable and health care professionals are trusted, patients are more likely to seek timely care.

Conversely, countries such as Nigeria and South Africa demonstrated a weaker association between trust and health care access delays, suggesting the importance of cultural, economic, and systemic factors in shaping individuals’ trust in their health care systems. This finding also highlights a potential for targeted interventions in these regions to build trust as a pathway to improving health care access.

Previous studies have found countries in Northern and Western Europe as well as Australia and New Zealand to have the highest rates of trust in scientists and health care professionals [24]. Countries in Central Africa and South America reported the lowest trust levels, which negatively affected COVID-19 messaging and response in countries such as Tanzania [29]. Additionally, there is evidence of an association between life expectancy, household income, and trust, with countries with higher life expectancies and individuals with higher incomes scoring higher on the trust scale.

This study’s findings reveal that trust in health care professionals significantly influences health care access patterns, even when controlling for demographic factors such as age, gender, education, and financial stability. This indicates that trust is a critical, independent factor affecting health care access, even beyond the barriers imposed by socioeconomic status or demographic characteristics.

### Limitations

While our findings indicate a significant association between trust in health care professionals and reduced delays in health care access, the cross-sectional nature of our study limits our ability to infer causality. Future longitudinal or experimental studies are needed to explore the directionality of this relationship and to identify the mechanisms through which trust may causally influence healthcare access.

Additionally, Self-reported data may introduce bias, and the sample may be more representative of individuals with higher socioeconomic status and access to social media. Future research should aim to include more diverse populations, particularly those most at risk of being underserved by health care systems. Lastly, the associations observed for covariates within our multivariable regression model should not be considered as direct effects on delayed health care access.

### Conclusion

Insights from this study serve as a call to action for health care professionals, policy makers, and researchers. Our findings suggest an association between trust in health care professionals and delays in health care access. However, establishing trust as a causal factor in improving health care access requires further research. Policymakers and health care providers should consider strategies to enhance trust as a potential avenue to improve health care utilization, while recognizing the need for additional evidence to support causality.

Efforts to rebuild trust must go hand in hand with initiatives aimed at mitigating economic disparities, enhancing health literacy, and ensuring food security. Transparent communication, community engagement, and public education campaigns must be central components of any strategy aimed at strengthening the patient–health professional relationship. Additionally, consistent quality and delivery of care within health systems can strengthen patient trust.

Health care systems have the opportunity to use the lessons learned from this study to foster an environment where trust can flourish. By doing so, they will not only enhance the quality of care but also promote equity in health care access. The road to recovery may be long, but by prioritizing trust and addressing the multifaceted needs of the population, health care systems can emerge from the pandemic more robust and more trusted by the people they serve.

## Data Availability

Deidentified participant-level microdata are available for download on GESIS.
