# Supplementary material for: Association Between Trust in Health Care Professionals and Health Care Access: Insights From an Online Survey Across 21 Countries
Source: Int J Public Health. 2025 Apr 10;70:1607884. doi: 10.3389/ijph.2025.1607884 (PMC12018240; doi:10.3389/ijph.2025.1607884)
Supplement: Supplementary file 1 [file Table1.DOCX]

Table 1. Unweighted survey characteristics

|  | Argentina | Brazil | Chile | Colombia | Egypt | Germany | India | Indonesia | Italy | Japan | Mexico | Nigeria | Peru | Philippines | Poland | South Africa | Spain | TÃ¼rkiye | United Kingdom | United States | Viet Nam |
| --- | --- | --- | --- | --- | --- | --- | --- | --- | --- | --- | --- | --- | --- | --- | --- | --- | --- | --- | --- | --- | --- |
| n | 12716 | 22115 | 12001 | 17942 | 30778 | 9354 | 40613 | 29571 | 17487 | 9978 | 22965 | 27358 | 16360 | 38977 | 16249 | 24982 | 9408 | 12859 | 8213 | 10629 | 25400 |
| age (%) |  |  |  |  |  |  |  |  |  |  |  |  |  |  |  |  |  |  |  |  |  |
| 18-24 years | 1710 (13.4) | 2490 (11.3) | 868 (7.2) | 4341 (24.2) | 8228 (26.7) | 305 (3.3) | 7458 (18.4) | 6516 (22.0) | 526 (3.0) | 130 (1.3) | 4767 (20.8) | 6525 (23.9) | 3646 (22.3) | 9781 (25.1) | 3062 (18.8) | 5320 (21.3) | 350 (3.7) | 604 (4.7) | 455 (5.5) | 674 (6.3) | 10077 (39.7) |
| 25-29 years | 1638 (12.9) | 2608 (11.8) | 1174 (9.8) | 2938 (16.4) | 6123 (19.9) | 657 (7.0) | 9546 (23.5) | 5980 (20.2) | 1190 (6.8) | 170 (1.7) | 3609 (15.7) | 6343 (23.2) | 2444 (14.9) | 6926 (17.8) | 1792 (11.0) | 4283 (17.1) | 694 (7.4) | 993 (7.7) | 638 (7.8) | 1025 (9.6) | 4127 (16.2) |
| 30-39 years | 3134 (24.6) | 5792 (26.2) | 2971 (24.8) | 4895 (27.3) | 8646 (28.1) | 2200 (23.5) | 14778 (36.4) | 9127 (30.9) | 3591 (20.5) | 753 (7.5) | 6343 (27.6) | 8631 (31.5) | 4020 (24.6) | 11547 (29.6) | 3427 (21.1) | 7720 (30.9) | 1967 (20.9) | 3310 (25.7) | 1787 (21.8) | 2747 (25.8) | 6513 (25.6) |
| 40-49 years | 2641 (20.8) | 5331 (24.1) | 2689 (22.4) | 2953 (16.5) | 4717 (15.3) | 2084 (22.3) | 5348 (13.2) | 5208 (17.6) | 4234 (24.2) | 2113 (21.2) | 4193 (18.3) | 3914 (14.3) | 2981 (18.2) | 6276 (16.1) | 2691 (16.6) | 4380 (17.5) | 2232 (23.7) | 3739 (29.1) | 1504 (18.3) | 2104 (19.8) | 2664 (10.5) |
| 50-59 years | 1808 (14.2) | 3573 (16.2) | 2233 (18.6) | 1731 (9.6) | 2024 (6.6) | 2224 (23.8) | 2125 (5.2) | 2133 (7.2) | 4084 (23.4) | 3139 (31.5) | 2632 (11.5) | 1444 (5.3) | 1843 (11.3) | 2861 (7.3) | 2023 (12.4) | 2005 (8.0) | 2136 (22.7) | 2429 (18.9) | 1694 (20.6) | 1777 (16.7) | 1162 (4.6) |
| 60-69 years | 1176 (9.2) | 1839 (8.3) | 1466 (12.2) | 851 (4.7) | 838 (2.7) | 1298 (13.9) | 902 (2.2) | 509 (1.7) | 2648 (15.1) | 2491 (25.0) | 1139 (5.0) | 427 (1.6) | 1068 (6.5) | 1252 (3.2) | 2032 (12.5) | 933 (3.7) | 1426 (15.2) | 1390 (10.8) | 1288 (15.7) | 1375 (12.9) | 650 (2.6) |
| 70+ years | 609 (4.8) | 482 (2.2) | 600 (5.0) | 233 (1.3) | 202 (0.7) | 586 (6.3) | 456 (1.1) | 98 (0.3) | 1214 (6.9) | 1182 (11.8) | 282 (1.2) | 74 (0.3) | 358 (2.2) | 334 (0.9) | 1222 (7.5) | 341 (1.4) | 603 (6.4) | 394 (3.1) | 847 (10.3) | 927 (8.7) | 207 (0.8) |
| edu (%) |  |  |  |  |  |  |  |  |  |  |  |  |  |  |  |  |  |  |  |  |  |
| College or more | 3079 (24.2) | 5807 (26.3) | 4500 (37.5) | 5962 (33.2) | 20626 (67.0) | 2472 (26.4) | 30313 (74.6) | 8139 (27.5) | 5081 (29.1) | 5178 (51.9) | 10150 (44.2) | 18231 (66.6) | 7211 (44.1) | 20636 (52.9) | 5196 (32.0) | 8956 (35.8) | 3459 (36.8) | 4304 (33.5) | 4743 (57.7) | 5820 (54.8) | 11288 (44.4) |
| Primary school or less | 3120 (24.5) | 8165 (36.9) | 1604 (13.4) | 2362 (13.2) | 1238 (4.0) | 481 (5.1) | 3298 (8.1) | 3793 (12.8) | 2032 (11.6) | 71 (0.7) | 1366 (5.9) | 706 (2.6) | 915 (5.6) | 2779 (7.1) | 1380 (8.5) | 1256 (5.0) | 2065 (21.9) | 2346 (18.2) | 342 (4.2) | 796 (7.5) | 1634 (6.4) |
| Secondary school | 6517 (51.3) | 8143 (36.8) | 5897 (49.1) | 9618 (53.6) | 8914 (29.0) | 6401 (68.4) | 7002 (17.2) | 17639 (59.6) | 10374 (59.3) | 4729 (47.4) | 11449 (49.9) | 8421 (30.8) | 8234 (50.3) | 15562 (39.9) | 9673 (59.5) | 14770 (59.1) | 3884 (41.3) | 6209 (48.3) | 3128 (38.1) | 4013 (37.8) | 12478 (49.1) |
| gender (%) | |  |  |  |  |  |  |  |  |  |  |  |  |  |  |  |  |  |  |  |  |
| Female | 5948 (46.8) | 10584 (47.9) | 6045 (50.4) | 8705 (48.5) | 12417 (40.3) | 5050 (54.0) | 18575 (45.7) | 13557 (45.8) | 9828 (56.2) | 4270 (42.8) | 11207 (48.8) | 9527 (34.8) | 7898 (48.3) | 19919 (51.1) | 8923 (54.9) | 13105 (52.5) | 5197 (55.2) | 5279 (41.1) | 4197 (51.1) | 6011 (56.6) | 11492 (45.2) |
| Male | 6570 (51.7) | 11391 (51.5) | 5789 (48.2) | 9021 (50.3) | 17943 (58.3) | 4195 (44.8) | 21804 (53.7) | 15654 (52.9) | 7422 (42.4) | 5580 (55.9) | 11362 (49.5) | 17708 (64.7) | 8309 (50.8) | 17970 (46.1) | 7125 (43.8) | 11585 (46.4) | 4110 (43.7) | 7491 (58.3) | 3913 (47.6) | 4386 (41.3) | 13344 (52.5) |
| Prefer not to answer or non-binary | 198 (1.6) | 140 (0.6) | 167 (1.4) | 216 (1.2) | 418 (1.4) | 109 (1.2) | 234 (0.6) | 360 (1.2) | 237 (1.4) | 128 (1.3) | 396 (1.7) | 123 (0.4) | 153 (0.9) | 1088 (2.8) | 201 (1.2) | 292 (1.2) | 101 (1.1) | 89 (0.7) | 103 (1.3) | 232 (2.2) | 564 (2.2) |
